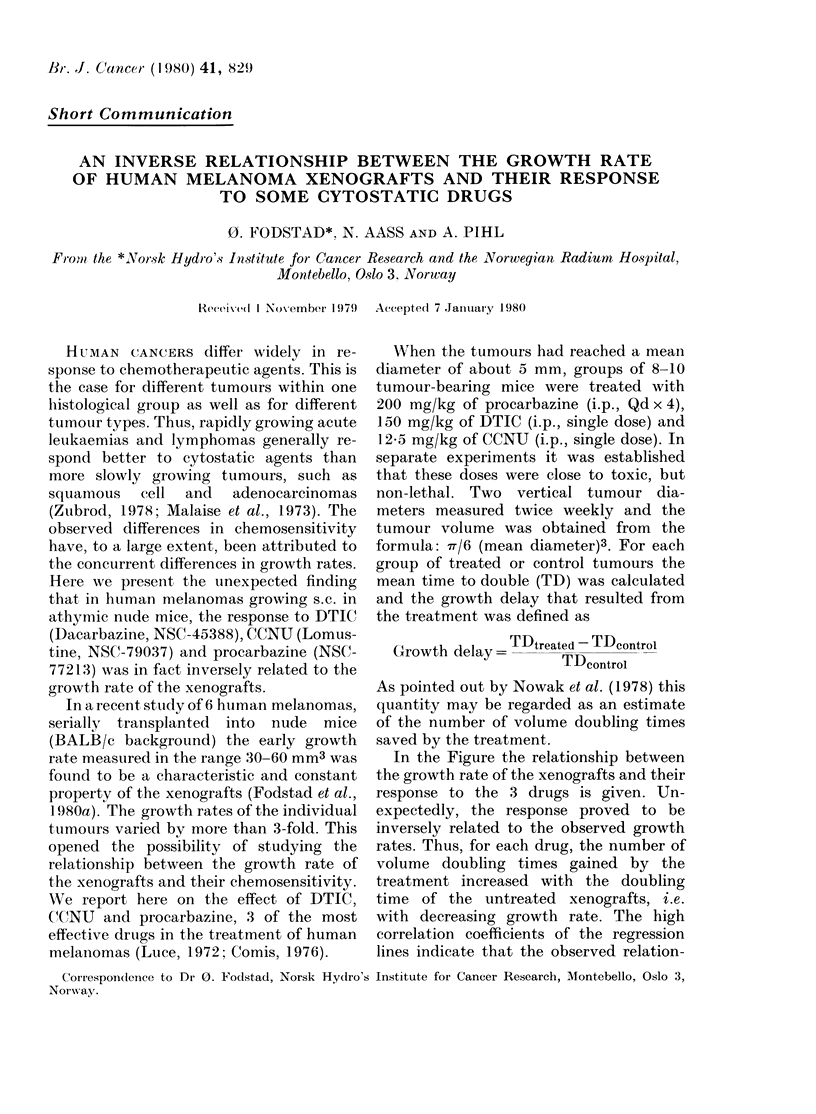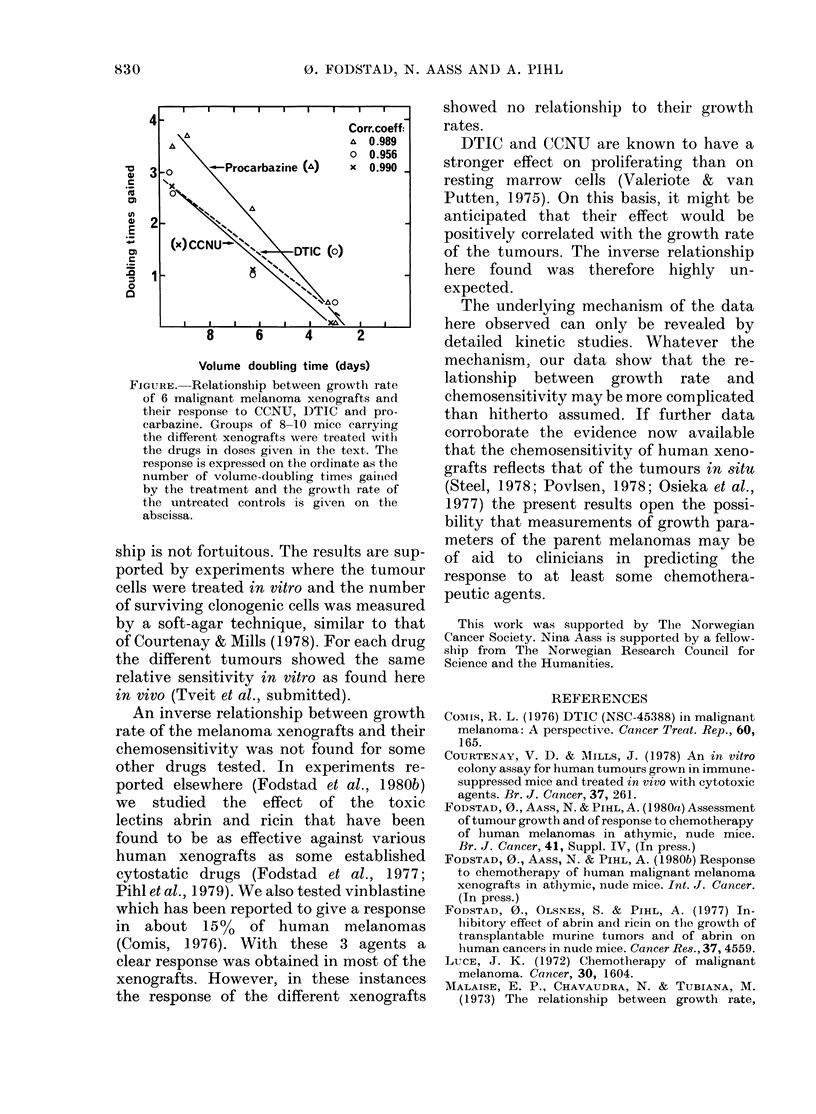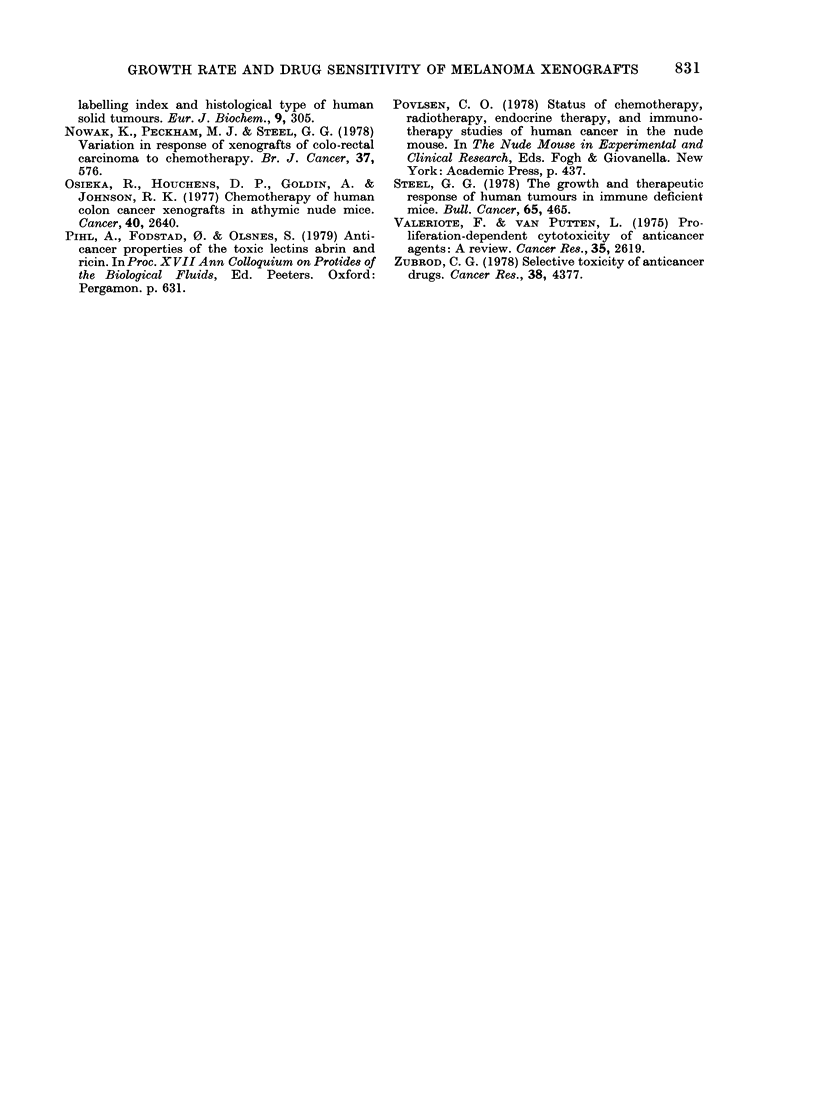# An inverse relationship between the growth rate of human melanoma xenografts and their response to some cytostatic drugs.

**DOI:** 10.1038/bjc.1980.149

**Published:** 1980-05

**Authors:** O. Fodstad, N. Aass, A. Pihl


*Br. J. Cancer* (1980) **41**, 829

***Short Communication***

### AN INVERSE RELATIONSHIP BETWEEN THE GROWTH RATE OF HUMAN MELANOMA XENOGRAFTS AND THEIR RESPONSE TO SOME CYTOSTATIC DRUGS

Ø. FODSTAD*, N. AASS AND A. PIHL

*From the *Norsk Hydro's Institute for Cancer Research and the Norwegian Radium Hospital, Montebello, Oslo 3, Norway*

Received 1 November 1979 Accepted 7 January 1980

HUMAN CANCERS differ widely in response to chemotherapeutic agents. This is the case for different tumours within one histological group as well as for different tumour types. Thus, rapidly growing acute leukaemias and lymphomas generally respond better to cytostatic agents than more slowly growing tumours, such as squamous cell and adenocarcinomas (Zubrod, 1978; Malaise *et al.*, 1973). The observed differences in chemosensitivity have, to a large extent, been attributed to the concurrent differences in growth rates. Here we present the unexpected finding that in human melanomas growing s.c. in athymic nude mice, the response to DTIC (Dacarbazine, NSC-45388), CCNU (Lomustine, NSC-79037) and procarbazine (NSC-77213) was in fact inversely related to the growth rate of the xenografts.

In a recent study of 6 human melanomas, serially transplanted into nude mice (BALB/c background) the early growth rate measured in the range 30–60 mm$^3$ was found to be a characteristic and constant property of the xenografts (Fodstad *et al.*, 1980*a*). The growth rates of the individual tumours varied by more than 3-fold. This opened the possibility of studying the relationship between the growth rate of the xenografts and their chemosensitivity. We report here on the effect of DTIC, CCNU and procarbazine, 3 of the most effective drugs in the treatment of human melanomas (Luce, 1972; Comis, 1976).

When the tumours had reached a mean diameter of about 5 mm, groups of 8–10 tumour-bearing mice were treated with 200 mg/kg of procarbazine (i.p., Qd $\times$ 4), 150 mg/kg of DTIC (i.p., single dose) and 12·5 mg/kg of CCNU (i.p., single dose). In separate experiments it was established that these doses were close to toxic, but non-lethal. Two vertical tumour diameters measured twice weekly and the tumour volume was obtained from the formula: $\pi/6$ (mean diameter)$^3$. For each group of treated or control tumours the mean time to double (TD) was calculated and the growth delay that resulted from the treatment was defined as

$$\text{Growth delay} = \frac{TD_{\text{treated}} - TD_{\text{control}}}{TD_{\text{control}}}$$

As pointed out by Nowak *et al.* (1978) this quantity may be regarded as an estimate of the number of volume doubling times saved by the treatment.

In the Figure the relationship between the growth rate of the xenografts and their response to the 3 drugs is given. Unexpectedly, the response proved to be inversely related to the observed growth rates. Thus, for each drug, the number of volume doubling times gained by the treatment increased with the doubling time of the untreated xenografts, *i.e.* with decreasing growth rate. The high correlation coefficients of the regression lines indicate that the observed relation-

Correspondence to Dr Ø. Fodstad, Norsk Hydro's Institute for Cancer Research, Montebello, Oslo 3, Norway.

FIGURE.—Relationship between growth rate of 6 malignant melanoma xenografts and their response to CCNU, DTIC and procarbazine. Groups of 8–10 mice carrying the different xenografts were treated with the drugs in doses given in the text. The response is expressed on the ordinate as the number of volume-doubling times gained by the treatment and the growth rate of the untreated controls is given on the abscissa.

ship is not fortuitous. The results are supported by experiments where the tumour cells were treated *in vitro* and the number of surviving clonogenic cells was measured by a soft-agar technique, similar to that of Courtenay & Mills (1978). For each drug the different tumours showed the same relative sensitivity *in vitro* as found here *in vivo* (Tveit *et al.*, submitted).

An inverse relationship between growth rate of the melanoma xenografts and their chemosensitivity was not found for some other drugs tested. In experiments reported elsewhere (Fodstad *et al.*, 1980*b*) we studied the effect of the toxic lectins abrin and ricin that have been found to be as effective against various human xenografts as some established cytostatic drugs (Fodstad *et al.*, 1977; Pihl *et al.*, 1979). We also tested vinblastine which has been reported to give a response in about 15% of human melanomas (Comis, 1976). With these 3 agents a clear response was obtained in most of the xenografts. However, in these instances the response of the different xenografts showed no relationship to their growth rates.

DTIC and CCNU are known to have a stronger effect on proliferating than on resting marrow cells (Valeriote & van Putten, 1975). On this basis, it might be anticipated that their effect would be positively correlated with the growth rate of the tumours. The inverse relationship here found was therefore highly unexpected.

The underlying mechanism of the data here observed can only be revealed by detailed kinetic studies. Whatever the mechanism, our data show that the relationship between growth rate and chemosensitivity may be more complicated than hitherto assumed. If further data corroborate the evidence now available that the chemosensitivity of human xenografts reflects that of the tumours *in situ* (Steel, 1978; Povlsen, 1978; Osieka *et al.*, 1977) the present results open the possibility that measurements of growth parameters of the parent melanomas may be of aid to clinicians in predicting the response to at least some chemotherapeutic agents.

This work was supported by The Norwegian Cancer Society. Nina Aass is supported by a fellowship from The Norwegian Research Council for Science and the Humanities.

REFERENCES

COMIS, R. L. (1976) DTIC (NSC-45388) in malignant melanoma: A perspective. *Cancer Treat. Rep.*, **60**, 165.

COURTENAY, V. D. & MILLS, J. (1978) An *in vitro* colony assay for human tumours grown in immune-suppressed mice and treated *in vivo* with cytotoxic agents. *Br. J. Cancer*, **37**, 261.

FODSTAD, Ø., AASS, N. & PIHL, A. (1980*a*) Assessment of tumour growth and of response to chemotherapy of human melanomas in athymic, nude mice. *Br. J. Cancer*, **41**, Suppl. IV, (In press.)

FODSTAD, Ø., AASS, N. & PIHL, A. (1980*b*) Response to chemotherapy of human malignant melanoma xenografts in athymic, nude mice. *Int. J. Cancer*. (In press.)

FODSTAD, Ø., OLSNES, S. & PIHL, A. (1977) Inhibitory effect of abrin and ricin on the growth of transplantable murine tumors and of abrin on human cancers in nude mice. *Cancer Res.*, **37**, 4559.

LUCE, J. K. (1972) Chemotherapy of malignant melanoma. *Cancer*, **30**, 1604.

MALAISE, E. P., CHAVAUDRA, N. & TUBIANA, M. (1973) The relationship between growth rate, labelling index and histological type of human solid tumours. *Eur. J. Biochem.*, **9**, 305.

NOWAK, K., PECKHAM, M. J. & STEEL, G. G. (1978) Variation in response of xenografts of colo-rectal carcinoma to chemotherapy. *Br. J. Cancer*, **37**, 576.

OSIEKA, R., HOUCHENS, D. P., GOLDIN, A. & JOHNSON, R. K. (1977) Chemotherapy of human colon cancer xenografts in athymic nude mice. *Cancer*, **40**, 2640.

PIHL, A., FODSTAD, Ø. & OLSNES, S. (1979) Anticancer properties of the toxic lectins abrin and ricin. In *Proc. XVII Ann Colloquium on Protides of the Biological Fluids*, Ed. Peeters. Oxford: Pergamon. p. 631.

POVLSEN, C. O. (1978) Status of chemotherapy, radiotherapy, endocrine therapy, and immunotherapy studies of human cancer in the nude mouse. In *The Nude Mouse in Experimental and Clinical Research*, Eds. Fogh & Giovanella. New York: Academic Press, p. 437.

STEEL, G. G. (1978) The growth and therapeutic response of human tumours in immune deficient mice. *Bull. Cancer*, **65**, 465.

VALERIOTE, F. & VAN PUTTEN, L. (1975) Proliferation-dependent cytotoxicity of anticancer agents: A review. *Cancer Res.*, **35**, 2619.

ZUBROD, C. G. (1978) Selective toxicity of anticancer drugs. *Cancer Res.*, **38**, 4377.